# Fighting mosquito bite during a crisis: capabilities of Florida mosquito control districts during the COVID-19 pandemic

**DOI:** 10.1186/s12889-021-10724-w

**Published:** 2021-04-08

**Authors:** Imelda K. Moise, Lola R. Ortiz-Whittingham, Vincent Omachonu, Marah Clark, Rui-De Xue

**Affiliations:** 1 Department of Geography, University of Miami, 1300 Campo Sano Ave, Coral Gables, FL 33124 USA; 2 Department of Industrial Engineering, 1251 Memorial Drive, Coral Gables, FL 33146 USA; 3 Florida Department of Agriculture and Consumer Services, 3125 Conner Boulevard, Tallahassee, FL 32399 USA; 4 Anastasia Mosquito Control District, 120 EOC Drive, St. Augustine, FL 32092 USA

**Keywords:** Fight the bite, Arbovirus, United states, GIS, Staffing, Survey, *Culex*, Zika, *Aedes*

## Abstract

**Background:**

The stay-at-home orders imposed in early April 2020 due to the COVID-19 pandemic in various states complicated mosquito control activities across the United States (US), and Florida was no exception. Mosquito control programs are the first line of defense against mosquito-borne pathogens. The purpose of this study was to examine the capabilities of Florida mosquito programs to implement key mosquito measures during the COVID-19 pandemic lockdown.

**Methods:**

Using a self-administered online survey, we examined the capabilities of all Florida mosquito control programs (both state-approved mosquito districts, *N* = 63; and open programs, *N* = 27) at a time when the state of Florida was still under heightened awareness of, stay-at-home orders and planning a phase 1 reopening over the COVID-19 pandemic (June to July 2020). The final sample included mosquito control programs structured as the Board of County Commissioners (BOCC) (*n* = 42), independent tax district (*n* = 16), municipal (*n* = 10), and health or emergency department (*n* = 5). We used descriptive statistics to summarize information about the characteristics of responding programs, their implemented mosquito control and surveillance activities.  wWe used bivariate analysis to compare the characteristics of responding programs and the self-reported mosquito measures.

**Results:**

Of the recruited mosquito control programs, 73 completed the survey (81.1% response rate; 73/90). Of these, 57.5% (*n* = 42) were Board of County Commissioners (BOCC) mosquito control programs, 21.9% (*n* = 16) were independent tax district programs, 13.7% (*n* = 10) were municipal mosquito control programs, and only 6.8% (*n* = 5) were either health or emergency department mosquito control programs. Except for arbovirus surveillance, most programs either fully or partially performed larval (61.8%) and adult (78.9%) surveillance; most programs conducted species-specific control for *Aedes aegypti* (85.2%, *n* = 54)*, Aedes albopictus* (87.3%, *n* = 55), *Culex quinquefasciatus* (92.1%, *n* = 58), and *Culex nigripalpus* (91.9%, *n* = 57)*.*

**Conclusions:**

Findings underscore the importance of ongoing mosquito control activities, and suggest that Florida mosquito control programs are vigilant and have significant capability to handle potential mosquito-borne disease threats, but arbovirus surveillance systems (laboratory testing of mosquito pools and testing of human and nonhuman specimens for arboviruses) are needed during pandemics as well.

## Background

Worldwide, mosquito-borne diseases continue to contribute to the global burden of infectious diseases, and produce epidemics of dengue, malaria, chikungunya, yellow fever and Zika that disturb health security and have wider socioeconomic impacts [1, 2]. Globally, mosquito-borne diseases account for more than 17% of all infectious diseases [3] and cause more than 700,000 deaths per year. In addition, mosquito-borne diseases pose a special challenge to public health practitioners and mosquito control districts [4–6] owing to their complex nature (biological transmission complexity) [7, 8] and potential to produce epidemics, particularly in areas that institutionally struggle to sustain mosquito management [9]. This makes surveillance and control key aspects for preventing mosquito-borne diseases and emerging arboviruses [4].

In Florida, *Aedes* and *Culex* continue to be major vector genera [10–12], with the state of Florida having been ground-zero for local transmission of Zika and dengue viruses [13]. The state is also in close proximity to Latin American where viruses such as Zika and dengue viruses are endemic [4, 14]. Therefore, surveillance as a key aspect of effective mosquito control and prevention [2, 15] is particularly important in economically depressed subtropical areas of the United States [1].

Now the stay-at-home orders imposed in early April 2020 in Florida due to the COVID-19 pandemic has complicated mosquito control activities [16], and raised questions about how we should manage mosquito control programs in the wake of pandemics or crises. The Centers for Disease Control and Prevention (CDC) underscore the importance of initiating or continuing the delivery of mosquito control and public health organization services during public health emergencies and responses to natural disasters in order to reduce the risk of mosquito-borne disease [17]. Despite the importance of mosquito control as a basic public health function, the National Association of County and City Health Officials (NACCHO) recently reported COVID-19 impacts on mosquito programs that operate under the auspices of local health departments [18].

This, coupled with major funding and capacity gaps, may put pressure on some already struggling programs, and may exacerbate timely and effective response to (re) emergent arboviral diseases in the future [9]. Therefore, to understand the challenges inherent in implementing mosquito activities during a pandemic, we assessed the capabilities of Florida state-approved mosquito control districts and open programs to carry out mosquito control activities at a time when Florida was still under heightened awareness of, stay-at-home orders and planning a phase 1 reopening over the COVID-19 pandemic. Our findings will shed light on the capabilities of Florida mosquito control programs, the first line of defense against mosquito-borne pathogens. It will also shed light on the challenges experienced by these programs to carry out mosquito control during a pandemic.

## Method

### Study design

In June 2020, a cross-sectional questionnaire-based internet survey was conducted using an anonymous electronic self-administered survey distributed to all Florida state-approved mosquito districts (*n* = 63) and open programs (*n* = 27) for 90 programs. A team from the University of Miami conducted the survey, at a time when the state was in a COVID-19 “full phase 1 re-opening plan” [19], and on a 2–3 month postponement of the arbovirus surveillance program as the state virus laboratory in Tampa was redirected for COVID-19 response.

### Survey instrument

The survey instrument was refined from previous similar studies to address the study objectives [9, 20, 21]. The survey was pilot tested with four mosquito districts and distributed using the online survey software Qualtrics. We obtained a list of program contact information from the Florida Department of Agriculture and Consumer Services (FDACS).

The questionnaire consisted of 35 questions divided into six sections: mosquito district characteristics (8 questions); staffing levels (4 questions); mosquito program capabilities and challenges (13 questions); program budgets (4 questions); COVID-19 communication (1 question); participant demographics and partnership needs (5 questions). Almost all but 10 questions consisted of closed-ended questions, which allowed respondents the opportunity to provide further detail if the ‘other, please specify’ option was selected from the multiple choices. The closed-ended questions were multiple choice, categorical, dichotomous and Likert-type questions with five-point rating scales.

#### Study population

We recruited all Florida mosquito control districts (*n* = 63) and open mosquito programs (*n* = 27) via email. They comprised of Board of County Commissioners (BOCC) mosquito control programs, independent tax district programs, municipal mosquito control programs, and either health or emergency department mosquito control programs. Representatives of mosquito control districts and open programs were contacted directly and were asked to complete the survey by July 6, 2020 (day survey closed). We sent weekly follow-up reminders during the first 2 weeks in June, and every 3 days during the third and fourth week. Follow-ups consisted of both email and telephone calls. Program respondent anonymity was maintained, and the researchers were blinded by using the web-based survey tool for data collection and collation.

#### Data analysis

Survey responses were analyzed using IBM SPSS Statistics, version 26.0 [18]. We used descriptive statistics to summarize information about the characteristics and implemented mosquito control and surveillance activities of responding mosquito control districts and programs. Bivariate analysis was used to compare the characteristics of responding mosquito control districts and programs and the self-reported mosquito measures performed. Characteristics of respondents’ mosquito program capabilities such as arbovirus, population, environmental surveillance and routine control of domestic mosquitoes were analyzed using the χ^2^ test or Fisher’s exact test.

## Results

### Mosquito district and program characteristics

**Fig. 1:**
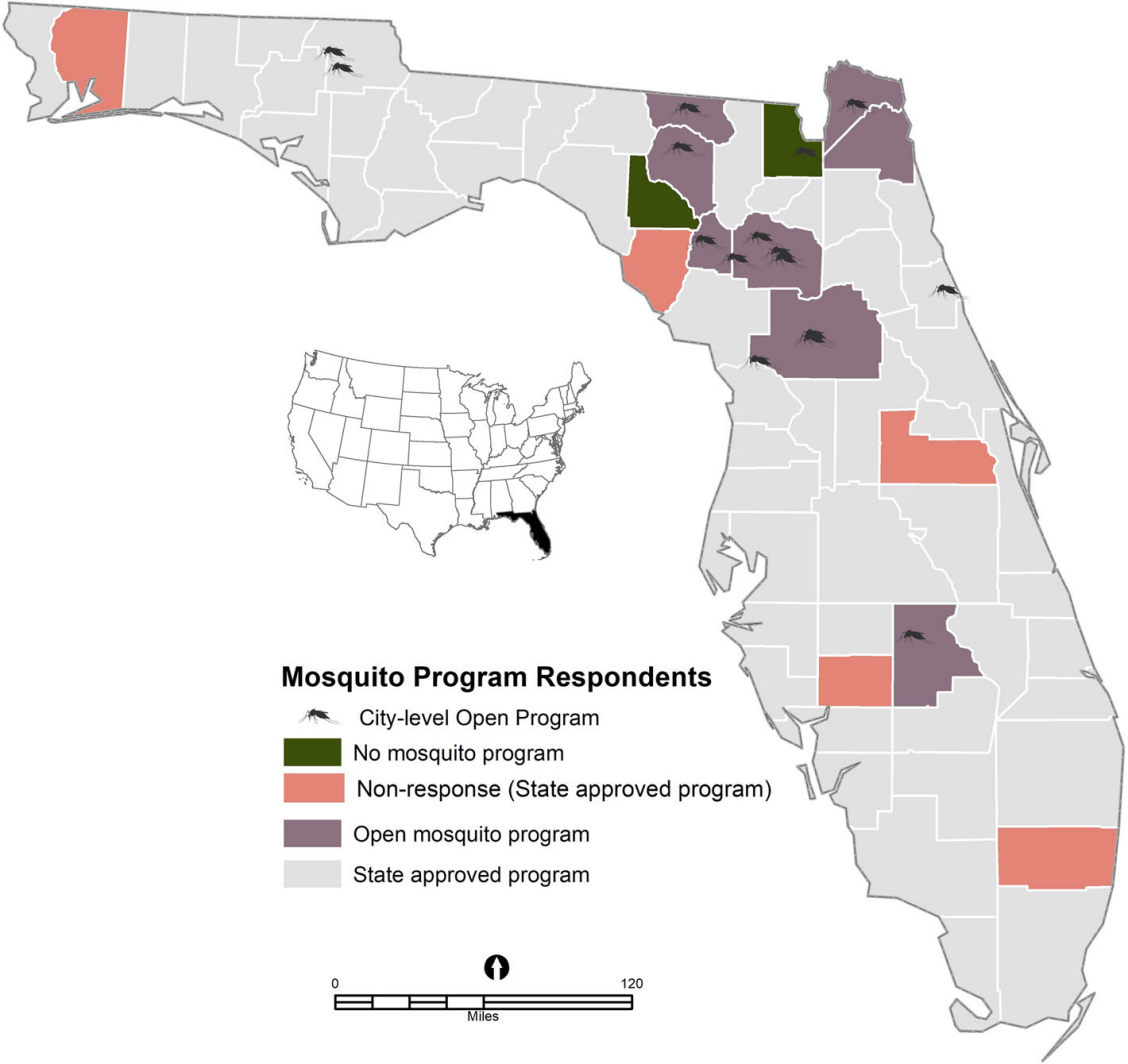
Map showing the locations of Florida Mosquito Control Districts and open programs that responded to the survey conducted in June 2020 by program type. Source: Map was generated by the first author

Of the recruited mosquito programs, 73 of 90 mosquito programs completed the survey (81.1% response rate). Five state-approved programs did not respond to the survey: one was due to the death of the mosquito director; one had the person responsible for mosquito activities reassigned to COVID-19 response, one did not have a person responsible for mosquito activities at the time of the survey and two did not respond. The excluded totaled four programs, including two that indicated not having a mosquito program (Baker and Lafayette Counties), and two with missing information on relevant measures. The final sample was 73 programs (58 state-approved mosquito control districts and 15 open programs) (Fig. 1).

Of the responding programs, 57.5% (*n* = 42) were Board of County Commissioners (BOCC) programs, 21.9% (*n* = 16) were independent tax districts, 13.7% (*n* = 10) were municipal programs, and only 6.8% (*n* = 5) were either health or emergency departments.

Nearly all responding programs (97.3%, *n* = 71 of 73) indicated performing mosquito control activities either fully or partially during the COVID-19 pandemic, and only 7.5% (*n* = 4 of 53) of programs that responded to the question “to what extent has COVID-19 affected your mosquito activities” reported being highly impacted by COVID-19. Three quarters of respondents (75.0%, *n* = 51) did not perform arboviral surveillance (send mosquito pools for testing). It seems possible that these results are due to the redirection of the state health laboratory in Tampa to COVID-19 response, and similarities of testing supplies needed for COVID-19 and mosquito pool testing (Table 1).

**Table 1:** Characteristics of responding mosquito control districts during the COVID-19 pandemic, Florida, USA, June 2020

	Number	Percent	95% CI
**Organizational Structure**			
Board of County Commissioners (BOCC)	42	57.5	(46.6–69.9)
Municipal	10	13.7	(6.8–21.9)
Independent Tax District	16	21.9	(12.3–31.5)
Health Department or other department	5	6.8	(1.4–13.7)
**Program Type**			
State-approved program	58	79.5	(69.9–89.0)
Open programs	15	20.5	(11.0–30.1)
**Did you operate during the pandemic?**			
Yes, fully open and operating	44	60.3	(49.3–72.6)
Partially operating with limited activities	27	37.0	(24.7–47.9)
No, closed operation until further notice	2	2.7	(0.0–6.8)
**To what extent has COVID-19 affected your mosquito activities?**			
High	4	7.5	(1.9–15.1)
Medium	12	22.6	(11.3–34.0)
Low	37	69.8	(56.6–81.1)
**Did you carry out non-chemical control activities?**			
Yes	37	54.4	(42.6–67.6)
No	29	42.6	(30.9–55.9)
Not sure	2	2.9	(0.0–7.4)
**Did you conduct pesticide resistance testing?**			
Yes, full capacity	11	16.2	(7.4–25.0)
No, we did not	51	75.0	(64.7–85.3)
Yes, limited capacity	4	5.9	(1.5–11.8)
Do not have a program or not applicable	2	2.9	(0.0–7.4)
**Will the pandemic affect your FY2020–2021 budget?**			
Yes	12	17.1	(10.0–25.7)
No	22	31.4	(20.0–42.9)
Not sure	36	51.4	(38.6–62.9)
**Can you hire interns/seasonal workers?**			
Yes	16	25.4	(15.9–36.5)
No	47	74.6	(63.5–84.1)
**Are staffing levels inadequate due to furloughs or lockdown?**			
Yes	8	12.3	(4.6–21.5)
No	57	87.7	(78.5–95.4)
**Did you communicate with state/local public health departments?**			
Yes	50	71.4	(60.0–81.4)
No	20	28.6	(18.6–40.0)

Excluded are four programs with missing data and those that do not have mosquito programs (e.g., Barker and Lafferty County). Health department includes emergency management programs

### Mosquito program capabilities for arbovirus, population, environmental surveillance

When asked whether the COVID-19 pandemic will affect mosquito control programs’ fiscal year (FY) 2020/2021 budgets, 82.9% (*n* = 58) indicated, “no” or that they were “not sure” (results not shown). There was also a large variation in the levels of main vector surveillance and control activities performed (Table 2). For example, while most mosquito control programs (both state approved and open programs) did not perform arbovirus surveillance using flocks of sentinel chickens (84.1%, *n* = 58) or mosquito pooling (83.8%, *n* = 68), the majority maintained larval and adult surveillance during the initial months of the COVID-19 pandemic (61.8%, *n* = 68 vs 78.9%, *n* = 71). More than three-quarters of mosquito control programs (70.8%, *n* = 65) did not conduct arbovirus surveillance using tidal surveillance, while 35 (49.3%) programs monitored temperature, wind and daylight. Of the responding mosquito programs, 36 (53.7%) used rain gauges for surveillance, *p* < 0.022. Climatic factors such as temperature, humidity, and rain have been linked to mosquito abundance and transmission [1, 22, 23].

**Table 2:** Arbovirus surveillance activities conducted during the COVID-19 pandemic, Florida, USA, June 2020

Did you conduct arbovirus surveillance during the COVID-19 pandemic using the following:	Yes, full or limited capacity n (%)	No, we did not conduct this arbovirus surveillance activity n (%)	***Fisher’s exact test***
			***P-***value
**Using flocks of sentinel chickens**			
Board of County Commissioners (BOCC) Programs	9 (21.4)	33 (78.6)	0.110
Other Mosquito Programs	2 (7.4)	25 (92.6)	
**Using mosquito pooling**			
Board of County Commissioners (BOCC) Programs	9 (22.0)	32 (78.0)	0.102
Other Mosquito Programs	2 (7.4)	25 (92.6)	
**Larval surveillance**			
Board of County Commissioners (BOCC) Programs	26 (61.9)	16 (38.1)	0.588
Other Mosquito Programs	16 (61.5)	10 (38.5)	
**Adult surveillance**			
Board of County Commissioners (BOCC) Programs	36 (85.7)	6 (14.3)	0.081
Other Mosquito Programs	20 (69.0)	9 (31.0)	
**With rain gauges**			
Board of County Commissioners (BOCC) Programs	17 (42.5)	23 (57.5)	**0.022**
Other Mosquito Programs	19 (70.4)	8 (29.6)	
**Tidal surveillance**			
Board of County Commissioners (BOCC) Programs	9 (23.1)	30 (76.9)	0.145
Other Mosquito Programs	10 (38.5)	16 (61.5)	
**Temperature, wind and daylight was monitored**			
Board of County Commissioners (BOCC) Programs	24 (57.1)	18 (42.9)	0.088
Other Mosquito Programs	11 (37.9)	18 (62.1)	

Other mosquito programs includes independent tax district, municipal and health department or other department mosquito programs

### Mosquito program capabilities for routine control of domestic mosquitoes

Despite the wide variation in performed mosquito activities, both state-approved and open programs either fully or with limited capacity performed species-specific control activities for *Aedes aegypti* (85.2%, *n* = 46)*, Aedes albopictus* (87.3%, *n* = 55), *Culex quinquefasciatus* (92.1%, *n* = 58), and *Culex nigripalpus* (91.9%, *n* = 57)*.* In some areas*, Aedes aegypti* has not been identified hence no control measures for this species were performed (eight BOCC mosquito control programs, one independent tax district and one health department program). Likewise, one independent tax district reported the same for *Aedes albopictus* and *Culex nigripalpus.* Except for rain gauge, a Fisher’s exact test of independence showed no statistically significant difference in the proportion of programs that performed mosquito measures by organizational structure (Table 3).

**Table 3:** Arbovirus control activities conducted during COVID-19 pandemic, Florida, USA, June 2020

Did you engage in routine control of these domestic mosquitoes during the COVID-19 pandemic?	Yes, full or limited capacity n (%)	No, we did not conduct this arbovirus surveillance activity n (%)	Species not identified in the area n (%)	χ^**2**^	***P-***value
***Aedes aegypti***					
BOCC Programs	28 (71.8)	3 (7.7)	8 (20.5)	3.373	0.185
Other Mosquito Programs	18 (72.0)	5 (20.0)	2 (8.0)		
***Aedes albopictus***					
BOCC Programs	34 (89.5)	4 (10.5)	0 (0.0)	1.889	0.389
Other Mosquito Programs	21 (80.8)	4 (15.4)	1 (3.8)		
***Culex quinquefasciatus***^a^					
BOCC Programs	35 (92.1)	3 (7.9)	0 (0.0)		0.666
Other Mosquito Programs	23 (92.0)	2 (8.0)	0 (0.0)		
***Culex nigripalpus***					
BOCC Programs	35 (92.1)	3 (7.9)	0 (0.0)	1.548	0.461
Other Mosquito Programs	22 (88.0)	2 (8.0)	1 (4.0)		

*BOCC* Board of County Commissioners; Other mosquito programs, includes independent tax district, municipal and health department or other department mosquito programs; ^a^Fisher’s exact test

## Discussion

Our findings suggest that despite the imposed COVID-19 lockdown or stay-at-home orders, the vast majority of responding Florida mosquito districts and open programs did not cease mosquito control operations. Those that remained open were mostly programs operating under the auspice of the Board of County Commissioners (BOCC), municipal and independent tax district. The mandate for these programs is to not only fight pest mosquitoes but also fight species of mosquitoes that have potential to transmit mosquito-borne pathogens [24, 25]. Florida’s mosquito control capabilities during the initial months of the COVID-19 pandemic may be attributed to the very nature of the ongoing mosquito control programs with permanent personnel, including the lessons learned from several prior research projects on mosquitoes and mosquito-borne diseases [26].

The observed lack of arbovirus surveillance for serology and pool testing is of primary concern as it limits the generation of evidence about when programs can anticipate a surge in arbovirus infection and in mosquito control programs’ capability to detect or monitor arbovirus presence for timely control response. To note, in Florida, most arboviral surveillance (serology and pool testing) is conducted at a state laboratory located in Tampa, a laboratory that was redirected to COVID-19 testing at the time of this study. This finding is not unique to Florida. The National Association of County and City Health Officials (NACCHO) reported similar challenges among county and city programs during the lockdown [20]. This is a persistent problem that was also reported by Hadler and his colleagues when they assessed “arbovirus surveillance 13 years after the introduction of WNV in the US” [21]. To prevent nuisance mosquitoes or diseases, there is a need for real time information. For example, if a mosquito is carrying Zika virus, we want to know that today, not in 2 weeks or months from now. The first author’s previous co-authored study identified over 1000 mosquito control agencies in the continental United States; 152 had publicly available open access mosquito datasets, and 148 agencies had live data that can be leveraged with good effect [27].

Notably, despite the observed marked differences in the level of performed mosquito control activities in Florida, most mosquito programs (both state-approved and open programs) performed mosquito control activities either fully or partially for key surveillance activities (Larviciding and adulticiding) during a time when the world was facing great challenges due to the COVID-19 pandemic. The majority of programs also engaged in routine control of domestic mosquitoes such as *Aedes* species of mosquitoes that can cause *Aedes*-borne arboviruses like dengue virus (DENV), chikungunya virus (CHIKV), yellow fever virus (YFV), and Zika virus (ZIKV) as well as *Culex* species of mosquitoes that can cause *Culex*-arboviruses like SLEV and WNV. In addition, mosquito surveillance is enhanced by the existence of ongoing meteorological, climatological, and water table monitoring [28]. This demonstrates that although Florida mosquito control programs have a long history and experience with the *Culex*-arbovirus systems, they are also capable of providing mosquito control against *Aedes* species as evidenced by the quick mitigation of the 2016 ZIKV outbreak [29].

### Limitations

The study has some limitations, which primarily stem from its cross-sectional and self-report survey design, a typical limitation of survey studies [30] as it can led to self-report bias.

However, when properly structured and implemented as was done in this study, self-reported responses provided valuable information on the views and opinions of mosquito programs regarding their capabilities to implement key mosquito measures to mitigate emergence and/or re-emergence of arboviruses. Moreover, the response rate was high (85.6%). In addition, we did not measure the capabilities of mosquito control programs from the perspective of residents or beneficiaries (e.g., whether the number of mosquitos or bites decreased or increased in Florida). This is an important issue for future research. We are also aware that other factors not directly examined in the study might also be important. For example, many environmental and geographical factors create differential vector densities and levels of human exposure [1, 6, 22, 31], resulting in differentiated surveillance and control needs [4, 9].

## Conclusion

Our findings have implications for local and state mosquito programs including national associations as they work towards mitigating the impacts of COVID-19. More importantly, maintaining sustainable systems for arbovirus surveillance for serology and pool testing is vital as it can help define the nature and extent of circulating strains of arboviruses, provide a basis for evaluating the risk of transmission of mosquito-borne diseases, contain a potential mosquito-borne disease outbreak and reduce the cost of responding to emerging vector-borne pathogens. This can also aid in gauging the efficacy of control activities. There is also a need to build the capacity of mosquito control district and program laboratories and for the establishment of viral genomic databases as a reference for current and future research.

## Data Availability

The datasets generated and/or analyzed during our study are not publicly available since the data could potentially identify some of the respondents when linked to other data but are available from the corresponding author on reasonable request.

## Abbreviations

FL: Florida
WNV: West Nile virus
BOCC: Board of County Commissioners
FDACS: Florida Department of Agriculture and Consumer Services
IRB: Institutional Review Board
CDC: Centers for Diseases Control and Prevention
